# Preface to special topic on Tri-Co Robots

**DOI:** 10.1093/nsr/nwad037

**Published:** 2023-02-16

**Affiliations:** Huazhong University of Science and Technology, China

The invention of the Unimate in the 1950s marked the landmark beginning of a 70-year boom in robotics. Today, the morphology, functionality and missions of robots have undergone revolutionary changes. Space robots taking over on-orbit services, surgical robots remotely controlled by doctors, dexterous wearable prosthetic hands, and robotic tools for manufacturing large parts have far exceeded expectations when industrial robots first emerged. There is an urgent need for new theories, methods, and technologies to meet these new trends. Summarizing the common features of the above scenarios, we propose the concept of ‘Tri-Co Robots’, which stands for Coexisting-Cooperative-Cognitive Robots, focusing on the basic theories and key technologies that enhance the robot’s capability of interacting with humans, the environment and other peer robots. In 2017, the National Natural Science Foundation of China (NSFC) launched the Tri-Co Robot major research program. Under this program, a number of funded projects have achieved remarkable progress in the past five years. In this special topic, we have selected a few representative works that demonstrate our perspectives on Tri-Co Robots and their future directions. It is worth noting, however, that we do welcome and look forward to a richer interpretation of Tri-Co Robots.

Robots have played important roles in manufacturing automation in the past; advances in robotics are transforming the paradigm of manufacturing today. Xie *et al*. report world-wide attempts on developing mobile robotic milling machines featuring a new manufacturing mode as ‘fixing the part, moving the machine’. To ensure the milling quality of large-scale, lightweight, and low-rigidity parts, dual-robot mirror milling has exhibited great potential and thus multi-robot cooperation and collaboration turn out to be an important topic. Climbing robots that can literally climb along large parts and conduct *in-situ* machining represent another type of ‘craftsmen’ robots for intelligent manufacturing. Climbing manufacturing robots, comprehensively reviewed by Tao *et al*., pose several key challenges regarding the adhesion principle, motion mechanism, positioning technology and control methods. Similarly, the utilization of multiple climbing robots for collaborative manufacturing is also an inevitable trend. To address the important challenges of robot-robot collaboration, Duan *et al*. review the collective behaviors of animals and the bioinspired swarm intelligence in cooperative cluster systems. They foresee the trend of adopting the cooperation of robotic systems to realize the multiplicative and exponential increase of the task capability.

Extreme environments impose challenging constraints on robot operation, and therefore, require robots with an enhanced ability to interact with the environment. Li *et al*. present the recent progress on space robots for on-orbit servicing, including well-established missions and ongoing proof-of-concept investigations. From search and rescue after earthquakes and nuclear explosions to extraterrestrial explorations, legged robots have the potential to outperform wheeled or tracked robots over irregular and harsh terrains. Gao *et al*. review the technical progress of multi-legged robots in mechanical design, actuators, controls, planning, and perception, as well as real-world applications in recent years, including their skiing and skating hexapod robots debuted in the Beijing 2022 Olympic Winter Games. In Ding *et al*.’s article, they propose an unsupervised learning framework for legged robots to learn the physical characteristics of terrains, endowing robots with better environmental adaptability. This work is an excellent example of showing how artificial intelligence plays a role in enhancing robot interactivity.

The development of human-centered robots, such as robotic prostheses and surgical robots, are growing rapidly nowadays. Gong *et al*.’s perspective discusses the principle-related and practical challenges regarding developing robotic prosthetic hands. Among many other challenges, the human-machine interface is critical for actual reconstruction of missing human limbs. Jiang *et al.* focus on recent progress in surface electromyogram neural-interfaces for intuitive prosthetic control in their review paper, and they believe affordable functional replacement for amputees is still a long way off. Bio-syncretic robots directly utilizing biological materials as their functional cores may benefit the robot's human-centered applications, due to their advantageous biocompatibility to avoid any immune response, as mentioned by Zhang *et al*. At the end of this special issue, in Li's interview with Prof. Guang-Zhong Yang, a medical roboticist, Prof. Yang shares his thoughts on Tri-Co Robots and their roles in medical treatment and rehabilitation.

Robotics is a rapidly-evolving field that requires a close collaboration over a wide spectrum of specialties, together with cooperation between academia and industry. With the deepening of such broader collaborations, we envision Tri-Co Robots taking over more challenging tasks in the near future.

